# Points

**Published:** 1898-07

**Authors:** 


### POINTS.

Glanders continues to be somewhat prevalent in certain districts of Indiana, and is causing much uneasiness among stock-owners.

A prominent St. Louis horse-dealing firm advised its buyers recently to leave the long-haired thin stock alone. Pity they were ever bred, for they have about as little substance as a turnip and about as much courage as a turkey.

Lieutenant-Colonel Smith, Assistant Quartermaster of the United States Army, purchased nearly a million dollars worth of mules the last week in May. It is estimated that $100,000 a day has been spent in these purchases since May 1st.

A noted New York veterinarian recently married a "sparrow," while a rising Philadelphia veterinarian recently tied himself up with a "bunch." Strange combinations in life are to be noted. What's in a name?

The *New York Medical Journal*, in an abstract from a foreign journal referring to the number of cases of persons treated at the Pasteur Institute at Odessa in 1895, numbering 1288; 284 of whom had been bitten by animals ascertained experimentally to be affected with rabies; 231 by animals recognized as rabietic by veterinary examination, and 663 by animals suspected to have rabies. Twenty-seven of the number bitten by animals shown to be rabietic had been bitten on the head and face. The creatures that had done the biting included 1 man, 1173 dogs, 43 cats, 2 horses, 16 wolves, and 3 hogs. Of the total number of persons treated five died of rabies.

The four hundred reindeer purchased by our Government arrived in New York safely, but with one loss while on shipboard. They were duly inspected by Dr. Hickman, of the port of New York, and sent on their journey to Puget Sound.

The Department of Agriculture is rapidly completing plans for the production of hog-cholera serum. The buildings and pens and preparation of the animals will soon enable the extensive experiments proposed to be undertaken.

The Canada Horse-breeders' Association will petition the coming Legislature of Ontario to establish an inspection for all horses standing for service in Ontario.

